# Obesity and intestinal stem cell susceptibility to carcinogenesis

**DOI:** 10.1186/s12986-021-00567-y

**Published:** 2021-04-07

**Authors:** Katayoun Pourvali, Hadi Monji

**Affiliations:** Department of Cellular and Molecular Nutrition, Faculty of Nutrition Science and Food Technology, National Nutrition and Food Technology Research Institute, Shahid Beheshti University of Medical Sciences, 1981619573 Tehran, Iran

**Keywords:** Obesity, High-fat-diet, Intestinal stem cell, Cancer, CRC

## Abstract

**Background:**

Obesity is a top public health problem associated with an increase in colorectal cancer incidence. Stem cells are the chief cells in tissue homeostasis that self-renew and differentiate into other cells to regenerate the organ. It is speculated that an increase in stem cell pool makes cells susceptible to carcinogenesis. In this review, we looked at the recent investigations linking obesity/high-fat diet-induced obesity to intestinal carcinogenesis with regard to intestinal stem cells and their niche.

**Findings:**

High-fat diet-induced obesity may rise intestinal carcinogenesis by increased Intestinal stem cells (ISC)/progenitor’s population, stemness, and niche independence through activation of PPAR-δ with fatty acids, hormonal alterations related to obesity, and low-grade inflammation. However, these effects may possibly relate to the interaction between fats and carbohydrates, and not a fatty acid per se. Nonetheless, literature studies are inconsistency in their results, probably due to the differences in the diet components and limitations of genetic models used.

**Conclusion:**

High-fat diet-induced obesity affects carcinogenesis by changing ISC proliferation and function. However, a well-matched diet and the reliable colorectal cancer models that mimic human carcinogenesis is necessary to clearly elucidate the influence of high-fat diet-induced obesity on ISC behavior.

## Introduction

The debate over cellular origin of cancer has always been controversial, and several theories have been put forward. One of the most recognized theories is the transformation of residing stem cells into cancer cells [1]; which is based on the assumption that cells need time to accumulate enough mutations to transform into a malignancy phenotype [2]. The adult stem cells located in tissues could obtain sequential mutations and pass them to next generation, which at a certain point triggers the stem cells to initiate uncontrolled proliferation and tumor formation [3]. In opposition, somatic cells, especially in the gut epithelium, generally have a short life span and are removed relatively quickly [4]. The oncogenic mutation accumulation may also occur in proliferating progenitor cells that could create cancer-initiating cells [2, 5, 6]. On the other hand, several similar cell signaling pathways have been identified in the normal stem cells and cancer stem cells, which have a critical role in the development of stem cells and cancer, including proliferation, self-renewal, migration, and differentiation [7].

Obesity is now recognized as an epidemic problem that imposes an enormous burden on human health in many countries. It is a risk factor for several diseases, including cardiovascular disease, rheumatoid arthritis, and type 2 diabetes mellitus [8]. Several common and non-common cancers are associated with obesity, such as breast cancer, thyroid cancer, prostatic cancer, and esophagus cancer [9]. Obesity also elevates the risk of colorectal cancer (CRC) by 30–70% in humans [10]. One of the primary contributions of obesity or high-fat diet-induced obesity to drive cancer formation is alteration of intestinal stem cells biology (ISCs). High-fat diet-induced obesity expands stem and non-stem cell pool, thereby, exposing them to more transformation [11]. The underlying mechanisms by which cells become susceptible to obesity-related carcinogenesis includes genetic factors, insulin/IGF-I resistance, chronic low-grade inflammation and oxidative stress, change in adipokines, and alteration in the microbiome [12–14]. Obesity/high-fat diet-induced obesity may also influence stem cells by regulating several signaling pathways and initiating or promoting cancer progress. The most important of this developmental signaling are notch, Wnt, EGF and BMP [15, 16], which are described later, in brief.

In the next sections, we will review obesity and high fat diet-induced obesity-associated modifications on intestinal stem cell (ISC) by focusing on underlying mechanisms. The role of obesity and high-fat diet components as potential extrinsic modulating factors of ISC signaling and a link between obesity/high-fat diet-induced obesity and intestinal tumorigenesis will be discussed.

## Intestinal stem cells

The intestinal epithelium is the first layer of both small and large intestine facing the gut tract lumen [17]. It is composed of a single layer of cells that is responsible for food digestion, nutritional uptake, and barrier preservation. It is the most self-renewing tissue of the body [18] that regenerated each three to seven days, depending on the species and anatomical region, achieved from a small population of intestinal stem cells (ISC) that reside at the bottom of the crypt of Lieberkühn [18–21]. The small intestinal architecture consists of crypts (proliferative compartment) and villus (differentiated compartment). Crypts include multiple stem cells, Paneth cells, enteroendocrine cells (EEC), and some goblet cells and the villi consists of maturated enterocytes as well as goblet and EEC [20, 22]. Proliferative ISCs are identified by expression of specific protein including, Lgr5 (leucine-rich repeat-containing G protein-coupled receptor 5), Ascl2 (achaete-scute complex homolog 2), also Olfm4 (olfactomedin 4) and low level of Sox9 (sex-determining region Y-box 9). Some researches provide documents supporting the presence of ISC population that appears to be more quiescent and could be activated during regenerative conditions. These quiescent (or reserve) ISCs are recognized by expression of Bmi1 (Bmi1 polycomb ring finger oncogene), Hopx (HOP homeobox), Lrig1 (leucine-rich repeats and immunoglobulin-like domains 1), and high levels of Sox9 mRNA [19, 23–27].

The intrinsic regulation by ISCs niche and the extrinsic signals from surroundings actively drive the cycling Lgr5 + ISCs to differentiate into proliferative intestinal cells [28]. Notch signals promote ISC maturation towards the absorptive specification once Notch is on or towards the secretory specification once off. Absorptive lineage, by default, differentiates into enterocytes, and becomes more mature while transferring upward through crypt-villi with BMP signals level increasing. Secretory progenitors differentiate to goblet, Paneth, or enteroendocrine cells (EEC). Once Notch and WNT pathway are off, the vast population of secretory progenitors become goblet cells. However, a high activity of Wnt signals along with fibroblast growth factor (FGF) prompts secretory progenitors toward Paneth cells. Low activity of Wnt and EGF and absence of Notch signals leads the EEC destination [29]. In the lack of Lgr5 + ISCs, the Bmi1 stem cells can maintain the stem cell pool [30].

ISC niche is a complex cellular construction that plays a vital role in ISC function. Lgr5 + ISC niche consists of Paneth cells in the small intestine [31], Reg4 + deep crypt secretory (DCS) cells in the colon, the extracellular matrix, mesenchymal pericryptal cells of lamina propria, immune cells, smooth muscle, endothelial and neural cells/nerve endings in lamina propria and submucosa cells of musclularis mucosae all over the small and large intestine [32, 33].These niche cells influence ISC function in part through regulating developmental signaling by secreting several growth factors and cytokine [31, 32]. Paneth niche cells support the proliferation and growth of Lgr5 + ISC through Wnt (Wnt3a), EGF, and Notch ligands (Dll4) expression in the small intestine. As Paneth cells do not exist in the colon, CD24 + -expressing cell, differentiated goblet cells, and DCS cells at crypt bottoms neighboring to Lgr5 + ISCs may play a similar role to Paneth cells in the small intestine and stimulate Notch signaling [34, 35].

Gut epithelial homeostasis is set by ISC self-renewal division and differentiation. It is speculated that more self-renewal increases stem cell population which can undergo a transformation and elevate the risk of tumor formation. On the other hand, declined self-renewal division or premature differentiation would diminish the ISCs population leading to a quasi-aging process that can also cause a malignancy [11, 36]. Several reports have indicated the role of LGR5 in cancer cell growth, metastasis, and sphere formation [37, 38]. Lgr5 + stem cell mRNA is more expressed in the crypt bottom during cancer growth [39, 40].

In Cancer Stem Cells (CSCs) Model, surviving, transformed subclones of tumor cells with SCs features drive tumor formation and progression. The similarity to normal tissue, in a cancer cell system, SCs-like cells divided into two cells, cancer stem cells and progenitor daughter cells, that began to proliferate and produce less stemness (nontumorigenic) cells [41]. Interestingly, mounting evidence shows that progenitor cells may at least partially revert to CSCs by some procedures such as Epithelial-Mesenchymal Transition (EMT) [42]. This process, characteristic of cancer cell plasticity***,*** resulted in cancer development. Shared signaling mechanisms between SCs and CSCs [1] include the Hedgehog, JAK/STAT, Wnt/β-catenin, SHH, Notch, P13K/PTEN, NF-*κ*B, and MAPK/ERK. In normal stem cells and CSCs, these signaling are common and engaged in the SCs division, self-renewal, migration, and differentiation [43]. The SCs pathways are changed in CSCs and are representative of the cancers.

In the tumor microenvironment, the niche signal similar to the SC niche has a critical role in acquiring more SC properties and plastic in cancer cells. CSCs alter their microenvironment and make a pathological tumor surrounding and associated hierarchy (pathological Stem Cell System) similar to the primary normal Stem Cell Systems (SCSs). The cancer is a highly heterogeneous clone related to the accumulation of gene mutations and epigenetic modifications to protect CSCs and progress them. Many of these mutations are engaged in the stimulation of self-renewal and stemness mechanisms in multiple ways [4, 44]. Unlike normal stem cells, these mutant self-renewal signalings in the CSCs have not only increased but are continually activated [45]. Reprograming these self-renewal and stemness pathways has extreme tumorigenic potential in tissue by producing a high cell turnover and progenitors' generation [44].

### Wnt signaling

Wnt signaling role has been well described in many stem cells and during cancer development, especially in colon. This pathway regularly subdivides into the canonical β-catenin (for cell fate determination) and non-canonical signaling (for regulating cell polarity) [46–50]. In the absence of Wnt ligand, glycogen synthase kinase 3 beta (GSK-3) is activated; therefore, β-catenin is phosphorylated and subsequently ubiquitinated and degenerated. Wnt ligand, which in the intestine is produced by Paneth and mesenchymal cells, binding to the receptor (e.g., ISCs) [51] stimulates signaling cascade by inhibiting beta GSK-3 [52]. Therefore, β-catenin is separated from the inhibitory complex (GSK-3) and enters the nucleus. In the nucleus, β-catenin binds to the T cell factor (TCF) transcription factor and regulates expression of target genes such as c-Myc, Nanog, Oct4 (octamer-binding transcription factor 4), and Sox2 (sex-determining region Y-box 2) [49]; thereby, maintaining proliferation, stemness, and structure of stem cells. Mutation of β-catenin or other Wnt components occur in many cancers [48, 53–55]. In mouse model, activation of Wnt pathway can initiate tumor formation and increase anchorage-independent cell growth in pancreatic cancer [56]. Activation of Wnt accompanied by NF-κB resulted in dedifferentiation of normal intestinal cells to stem cells and consequently, tumor formation [56]. Over-activation of β-catenin resulted in proliferation and increase of sphere formation in tumor cells and induced the CSC phenotype in more differentiated cells [56]. Wnt signaling also induced expression of the TERT gene and resulted in increased telomerase activity and by that maintained the self-renewal and stemness properties [57].

### Notch signaling

Notch signaling is considered as a conserved evolutionary pathway that is involved in cell fate determination, proliferation, self-renewal, differentiation, and hemostasis in many cells [58]. The Notch signals have a unique character in developmental pathways as they can affect neighboring cells, so that Notch signal in one cell can modulate the fate of an adjacent cell [59, 60]. The Notch signaling pathway consists of notch receptors and Notch ligands that are single-pass transmembrane proteins with three domains (extracellular, transmembrane, and intracellular). Notch receptors have four different receptors (e.g., NOTCH 1–4), while Notch ligands have five ligands (e.g., Delta‐like (DLL) 1, 3, 4 and Jagged (JAG) 1, 2). Notch signaling is the interaction between juxtaposed cells; ligand expressing (the signal-sending, e.g., Paneth cells and proximate ISCs or progenitor cells in the intestinal crypt) and receptor‐expressing (signal‐receiving; e.g., ISCs) cells [59, 61]. Proteolytic cleavage of notch receptor is induced which in turn releases Notch intracellular domain (NICD); it is translocated to the nucleus and (1) in the canonical pathway: NICD interacts with the DNA-binding protein RBPJ (Recombination signal binding protein for immunoglobulin kappa J region) and the co-activator Mastermind (Mam; Mastermind-like transcriptional co-activator 1 (MAML1) to activate transcription of target genes including HES-1, -5, -7, HEY-1, -2, and HEYL genes encoding basic helix-loop-helix/orange domain transcriptional repressors and c-myc, or (2) in the non-canonical pathway, NICD together with p50 or c-Rel stimulates NFκB activity [15, 61–63].

Notch molecular signaling has an essential function in the CSC or cancer-initiating cell. It is shown that Notch signaling is more activated in CSCs compared to regular cancer cell lines [15]. Notch induces the initiation of colon cancer but does not affect the progression of it in the mouse model [64]. In human colon cancer, the Notch pathway is highly expressed in adenomas and CRCs during the initiation stage, but not expressed in advance and aggressive CRCs. The inhibition of Notch pathway increases chemoprevention and decreases sensitivity to the therapy [65].

### Epidermal growth factor (EGF)

EGF is a small mitogenic protein that stimulates the proliferation of CBCs and guts epithelial cells all over the crypt-villus axis. In the intestine, EGF and transforming growth factor- α (TGFα) are expressed by Paneth cells and mesenchymal cells such as enteric glial cells. The EGF receptor (EGFR), which is a receptor tyrosine kinase, is vastly expressed in ISCs [29, 66].

High activity of EGF signaling under a Kras mutant stimulates ISC proliferation and is associated with stem cells' predominance in the epithelium. Since this overactivity of EGFR signaling is related to a high occurrence of neoplastic lesions, the signaling is closely regulated. In view of that Lrig1, which is greatly produced by CBCs, act as negative regulation of the EGFR and controls the hemostasis of the CBC milieu by modulating the growth factor signaling [66, 67]. Knockout of Lrig1 in mice leads to enlargement of the gut due to dramatic epithelial growth. This high growth is an outcome of unrestrained EGF signaling and demonstrates the importance of the signaling in regulating intestinal epithelium hemostasis [29, 68].

However, it is not fully understood how LRIG1 inhibits EGF pathways. While blocking of EGFR signaling abolishes the division rate of ISCs and induces quiescence in these cells, in vitro, the cells are capable to self-organize and re-enter the cell cycle once EGF signaling is retrieved. This feature discriminates EGF from Notch and Wnt pathways, removing these signaling accompanied by loss of stem cell identity [29].

### BMP and BMP antagonists

BMPs are multi-functional cytokine that is a member of the TGFβ superfamily of proteins [69]. When BMPs bind to their type II receptors recruit and stimulate type I BMP receptors, sequentially, regulate mothers against decapentaplegic homologue 1 (SMAD1), SMAD5 or SMAD8, with the co-mediator SMAD4 and accumulate to the nucleus, then modulate target gene transcription [70]. In the gut crypt, mesenchymal and stromal cells secret BMP2 and BMP4 as the main ligands for Bmpr1a (main BMP receptors in the intestine) [29, 71, 72]. BMPs induce the proliferative cues from the niche to promote differentiation of ISC and progenitors. BMP pathways need to be exactly tuned to make a balance between expansion at the base of the crypt and differentiation of the compartment of differentiated cells above it. For example, a high BMPs activation in the stem cell compartment could rapidly diminish epithelium. Thus, some niche cells at the basal crypt, such as myofibroblasts and smooth muscle cells, secrete BMP antagonists like Noggin, Follistatin, Chordin, and Gremlin, which make a BMP-low milieu at the base of the crypt toward a BMP-high milieu at the top of villi [29, 73, 74].

Inhibition of BMP pathways promotes the stem zone with cumulating Wnt signaling and finally leads to polyp formation. The MOB1A/B of the Hippo signaling integrated with BMP/TGF-signaling to control Wnt activity in the epithelium and regulate their homeostasis. These findings reveal the interaction between Wnt, Hippo, and BMP/TGF- signaling in crypt cells for determining intestinal epithelial fate [66]. More studies discovered that the BMP pathway inhibition stimulates PI3K-AKT signaling, accumulation of β-catenin into the nucleus, and increased self-renewal of ISCs [71].

### Obesity, intestinal stem cell and cancer formation

The scientific evidence that link obesity or HFD-induced obesity involvement in molecular signaling regulation of intestinal stem cells, with colon cancer is relatively little. However, such researches are increasing (Table 1) to reveal the impact of obesity and high-fat diet-induced obesity on ISCs. For instance, the hyperphagia db/db obese mouse model and high-fat diet-induced obesity increased villus height, crypt depth, and ISC numbers and proliferation in mice [22, 75]. Other studies indicated that HFD increased small and colon ISC numbers, thereby predisposed them to cancer [22, 76–81]. In addition to these quantitative (or number of ISCs) alterations, several studies also uncovered qualitative (stemness and self-renewal properties) changes in ISC. For instance, Beyaz S. et al. showed that mouse non-stem progenitor intestinal cells gain stemness (increased organoid-initiating capacity) and self-renewal properties with the high-fat diet, showing the potential of progenitor cells to acquire stemness properties and transform to cancer-initiating cells in response to high-fat diet-induced obesity [77].

**Table 1:** Effect of high-fat diet-induced obesity on intestinal stem cells

	Intervention	The effects on ISC	Mechanism	References
1	Sox9-EGFP mice, low-fat chow (14% kcal from fat) or HFD (45% kcal for 20 weeks Jejunum	Increases ISC number and proliferation, decreased Paneth and goblet cell numbers, no change in EEC	Correlated with insulin or IGF1 signaling impairment	[22]
2	High-fat diet induced obesity, independent of the high-fat diet	Increased crypt depth, villus height, the number of intestinal epithelial stem cells and goblet cells in vivo	-	[81]
3	HFD (60% fat) for 9–14 months), vs standard chow-fed counterparts	Non-stem progenitor intestinal cells gain more stemness features and self-renewal Lgr5 + ISC numbers in the small intestine and colon was increased Villous enterocyte numbers was decreased A 50% increase in the number of Olfm4 + ISCs and 23% decrease in niche Paneth cell numbers Crypts from the small intestine and colon were further likely to initiate mini-intestines in culture than those from controls	Induce in b-catenin target genes Jag1 and Jag2 (both ligands for Notch signaling) PPAR-δ signaling	[77]
5	male Lgr5 + -GFP fed with HFD (45% fat) vs purified sucrose-matched LFD (10%) for 12 months	Obesity did not effect on ISC proliferation and related pathways (Akt, MAPK, and Wnt) of Lgr5 + ISCs Pten inactivation alone, or combined with obesity, is insufficient to drive Lgr5 + -ISC-derived tumorigenesis	HFD upregulate fatty acid metabolism and PPAR signaling	[91]
6	Wild type male C57BL/6 J mice HFD (60% fat) in AOM injection (an initiation) model of colorectal cancer vs LFD (10% fat)	Increased aberrant crypt foci (ACF) Zone of proliferation in the HFD group was significantly larger than the LFD group		[143]
7	Lgr5-EGFP-IRES-creERT2 transgenic mice. HFD (60% fat) or low fat (10% fat) diet (Research Diets,) for 12 weeks	Higher number of Lgr5-GFP + stem cells per crypt	Adiponectin signaling	[80]
8	Female mice HFD (60% fat), 20% as carbohydrate and 20% as protein for 14 weeks	In the colon, the length was significantly reduced. Crypt deep decreased. Number of goblet cells decreased. the ISC numbers was increased but crypt function not changed In small intestine, villi length decreased. Number of ISCs and progenitor cells was increased. And crypts were further likely to form mini‑intestine organoids in a 3D culture Barrier function of the small intestine is not altered by HFD. The proportion and count of Paneth cells was not altered in the small intestinal crypt derived from organoids	Possibly the inflammatory factors and monocyte chemoattractant protein‑1 (MCP‑1)	[79]
9	HFD in Drosophila	Induced a transient activation of intestinal stem cells by microbiota	Induces JNK signaling in enterocytes, which triggers production of the cytokine upd3, thereby activates STAT signaling in intestinal stem cells	[78]
10	Hyperphagia db/db obese mouse model and a HFD-induced obesity mouse model fed with HFD (60% fat) for 8 weeks vs a standard chow diet	ISCs division, villi length, and nutrient absorption increased in both models	Upregulation of b-catenin protein along with inactivation of glycogen synthase kinase (GSK)-3b and Cyclin-D1, independent of leptin signaling	[75]
	HFD (23% fat) vs 5% fat in control feed in a pig model	Expanded colon stem cell zone and proliferative zone earlier onset of obesity and insulin resistance. induce inflammation in Proliferative zone, but not the stem cell zone	the increase in inflammation mediators (TLR-4, NF-kB, LCN-2 and IL6) induced with intestinal bacterial flora dysbiosis	[125]
11	Lgr5-EGFP-iresCreERT2 male and female mice. HFD (60%) vs 10% LFD fed for 3 months	HFD induced similar effects on early growth of ISCs in males and females. The ISCs from females showed a greater growth that was independent of obesity and sex steroid hormones The diet, sex and interaction between diet and sex does not affect lysozyme (Paneth cell marker) and mucin 2 (goblet cell marker). The Lgr5 (IESC marker) expression was upregulated in females than male independent of diet or interaction between diet and sex	The effect not related to sex steroid hormones	[135]
12	WSD (Fat 20% with a low vitamin D concentration compare 5%) for 3 months	The number of LGR5-ISCs was decreased and the Bmi + ISCs was increased. A complex transcriptional reprogramming including a mutational signature characteristic of replicative damage of human tumors was induced in both stem cell populations	Lower intake of vitamin D3 and/or calcium	[139]
13	a chow diet or a 1.25% cholesterol diet	Improve ISCs function	Modulating the levels of dietary cholesterol, which mediates phospholipid remodeling and tumorigenesiss	[82]
14	HFD independent of obesity in a xenograft model of colon cancer	Induce LGR5 expression, stem cell transformation, and colon carcinogenesis	Through a vitamin A-bound serum retinol binding protein 4-stimulated by retinoic acid 6 (RBP4-STRA6) signaling pathway	[136]
15	HFD in mice with an APC mutation,	Induce proliferation and DNA damage in Lgr5(+) cells. On the other hand, selective activation of intestinal FXR decrease abnormal Lgr5(+) cell proliferation	Increased levels of bile acids causing the repression of farnesoid receptor X (FXR), a sensor of nutritional cues in ISCs	[87]

Obesity could affect the function of intestinal stem cells, independent of high-fat diet components. Nutrient-sensing signaling, inflammatory signaling pathways, and hormonal signaling activated by high-fat diet components and obesity are involved in altering the function of intestinal stem cells [51]. In this regard, evidence shows that dietary cholesterol could regulate ISCs proliferation and differentiation [82]. Dietary protein is the main regulator of gut metabolism. However, it is currently unknown whether specific dietary amino acids directly modulate ISC behavior. It is demonstrated that Flies consumed a common food enriched with 1% glutamate have elevated growth of the small intestine and stimulates ISC division by the metabotropic glutamate receptor mGluR, a G-protein coupled receptor that causes elevation in intracytoplasmic Calcium2 + via phospholipase C and IP3 [51, 83]. An elevation of cellular Calcium2 + is a typical way of different mitogenic and stress pathways modulating ISC division, including insulin/IGF-1, Notch, and exposure to bleomycin. These results show that constant glutamate or Calcium2 + signaling can result in decontrolled ISC division, increasing the risk of tumorigenesis [51].

Calorie intake and amino acids leucine, glutamine, and arginine affects ISCs by regulating mTORC1 (mechanistic target of rapamycin complex 1) in the crypt Paneth cells. Calorie restriction reduced mTORC1 signaling in the Paneth cells, results in stimulation of the bone stromal antigen 1 (Bst1)—a lipid-anchored ectoenzyme that generates cyclic ADP ribose (cADPR). cADPR activates a NAD-dependent deacetelyase sirtuin 1 (SIRT1), in adjacent ISCs. Subsequently, SIRT1 phosphorylates mTORC1, fundamentally increasing mTORC1 activity in ISCs, which leads to self-renewal of ISCs [84, 85]. cADPR also stimulates calcium signaling in ISCs, which increases ISC proliferation. In addition, food consumption or insulin administration stimulated mTORC1 in the intestinal Paneth cells but not in ISCs in a rapamycin-sensitive manner. These documents proposed that Paneth cells can regulate ISC behavior by sensing the nutritional clues through mTORC1 [84, 85]. Prospect researches are needed to understanding the function of mTORC1 in the interaction between microenvironmental cells and SCs and how environmental clues modulate mTORC1 activity in these cells [85].

In Drosophila, food cholesterol modulates the differentiation of ISCs by modifying the Notch pathway, which results in the proliferation of secretory entero-endocrine cells in the epithelium [86]. HFD changes bile acids in the intestine lumen, which initiate the transformation of ISCs to a malignant phenotype by dysregulating Wnt pathway [87]. Moreover, the fatty acid composition of the high-fat diet increased the self-renewal potential of organoid bodies and enhanced stem cell acquisition in progenitor cells through PPAR-δ activation [77]. However, some studies uncovered the effects of obesity, independent of the high-fat diet components, on the quantity and quality of intestinal stem cells. For example, DeClercq and coworkers demonstrated that high-fat diet-induced obesity increases Lgr5-GFP + cell proliferation and numbers through adiponectin signaling [80], as well as inducing prolonged impacts on the proliferation of ISC, expanded crypt depth, villus height, numbers of gut stem cells, and goblet cells in mice [80]. It also increased the size of the entero-spheres in vitro [81].

Although the molecular mechanisms linking obesity to loss of ISCs function with the expansion of colon tumorigenicity remains to be fully elucidated, current literature recommends several mechanisms. Activation of PPAR-δ by FA components of a high-fat diet, hormonal imbalance related to obesity including insulin/IGF-1 resistance and adiponectin decline, increase in inflammation, and metabolic alteration by high-fat diet-induced obesity and microbiota dysbiosis could be named [88].

### PPAR- δ activation with fatty acid

PPAR-δ is a key regulator participating in fatty acid oxidation [89]. Several studies on the role of PPAR-δ in intestinal carcinogenesis propose that PPAR-δ acts as a transcriptional target for the Wnt/β-catenin signaling [77, 90]. In mice treated with high-fat diet or a PPAR-δ agonist, WNT/β-catenin pathway was activated and formed organoids, indicating a stemness acquisition and tumor-initiating potential in response, in a subset of non-ISC progenitors (but not terminally differentiated cells). Furthermore, the authors indicated that the sensitivity of ISCs to the Wnt/b-catenin pathway was elevated, thereby sustained more stemness in the crypt. They demonstrated that a high-fat diet stimulated PPAR-δ pathway and promoted Wnt target genes expression (Jag1, Jag 2 and Bmp4) in ISCs and progenitors. Jag1 and Jag 2 are ligands for the Notch pathway and are expressed by neighboring Paneth cells, which mediate juxtacrine Notch signaling in nearby ISCs to inhibit the secretory cell differentiation. Following decrease of Paneth cells with a high-fat diet, proximate ISCs or progenitor cells would alternatively provide Notch ligands [77]. These researchers provide documents that show fatty acid drive ISCs to niche independence**.** An increase in PPAR-δ expression with a high-fat diet in ISC was also observed in another study [91]. Two main pathways have been suggested regarding the transfer of PPAR-δ ligands to the nucleus. Firstly, fatty acids can transport to the nucleus by fatty acid-binding proteins (FABPs). In the Second way, ligands are formed in the nucleus as a product of lipid metabolism [77].

In addition, It is uncovered that Lgr5 receptor directly affects Wnt signaling pathway [92]. The mechanism proposed involves binding of LGR5 to R-spondin in stem cells, which increases the internalization of the frizzled- Wnt-LRP6 into the cell, leading to induced phosphorylation of Lrp6, and consequently increased β-catenin activity [93].

Together, these documents support the point that fatty acid component of a high-fat diet gives rise to ISC proliferation, Paneth niche cell independence, and stemness characteristic in gut progenitors through activating Wnt/β-catenin pathway downstream of PPAR-δ [51]. Further research is needed to disclose whether PPAR-δ activation by various high-fat diet components, a ketogenic diet, or prolonged obesity are associated with tumor initiation.

### Hormonal alteration associated with obesity

Obesity is associated with an imbalance of endocrine hormone signaling, especially impairments in insulin/IGF-1 pathway, decrease of adiponectin signaling, and leptin over-secretion and resistance.

#### Leptin

Obesity is associated with abundant circulating leptin; however, it stimulates numerous cellular processes that attenuate sensitivity to leptin leading to development of a metabolic disorder, known as leptin resistance [94]. Leptin is acts as a growth factor for many tissue such as the mammary gland, lung, liver, and colonic epithelium [95, 96]. It is shown that leptin stimulates proliferation of breast non-neoplastic cells and increases stem/progenitor numbers leading to a higher chance of cancer development. The elevated leptin disrupt cell polarity by overstimulation of the PI3K/Akt signaling. These effects are associated with increased tumorigenesis in breast tissue [97]. However, concerning the colon cancer, it is demonstrated that high-fat diet in the ob/ob mice, with a lack of leptin receptor, increased ACF multiplicity, as an early indicator of tumorigenesis [98]. Likewise, in a genetic mice model with leptin receptor knockout, high-fat diet increased ACF numbers [99]. The leptin receptor–deficient (*db/db*) mouse is a model of obesity that mice developed obese on chow or control food, not HFD. Interestingly, it is demonstrated that gut alteration in these mice is unlike to what is occurred in HFD-treated group [77]. For example, *db/db* mice fed chow show larger small intestinal mass and size, smaller crypts, and extensive villi compare to their wild-type mice. The quantities of ISCs and also Paneth cells are decreased; however, stem cell function is similar to control group. Lastly, PPAR-δ and Wnt/β-catenin signaling pathways are not altered in the ISCs and progenitors [51, 77].

Furthermore, although leptin receptor expresses in colon epithelial cells [95], it was not expressed in the colonic Lgr5 + stem cells and treatment with leptin did not affect numbers of Lgr5-GFP + stem cells [80]. Nevertheless, researchers demonstrated the roles of leptin receptor signaling in CRC development mediated via stimulation of STAT3 signaling [99, 100]. Collectively, these documents propose that leptin may play a small role in intestinal stem cell function and early stages of tumor formation, but possibly are involved in CRC progress.

#### Insulin/IGF-1

The insulin/IGF-1 signaling pathway may be involved in modulation of intestine epithelium hemostasis following obesity. Obesity is associated with high concentrations of circulating insulin and IGF-1 secreted from the pancreas and hepatic tissue. Obesity is also associated with elevated levels of local gut IGF-1 secreted from the mesenchymal cells around the ISCs [101]. Insulin receptors (A and B) as well as IGF-1 receptors are produced in ISCs. Elevated circulating and local levels of insulin and IGF-1 in the ISC microenvironment induce proliferation of ISCs [22]. Furthermore, the blood concentration of insulin and IGF-1 are significantly associated with increased colorectal cancer incidence, independently of weight and environmental factors [51, 102]. The IRS1 is markedly greater produce in CRC cells than normal mucous, proposing that the insulin pathway act as a stemness and cancer-initiating signaling, parallel to its function in stem cells [22, 103].

Mah et al. [22] demonstrated HFD-induced obesity enhanced proliferation and self-renewal of Sox9-EGFP^Low^ ISC, which are enriched for the Lgr5, and reduced numbers of Paneth and goblet cell in mice. Obtained ISC from HDF-treated mice produce fewer enteroids, indicating impaired ISC function. However, this reduced enteroid formation was reversed by insulin and IGF1, proposing that insulin/IGF-1 signaling mediates stemness acquisition of ISC in HFD-induced obesity [22]. Besides, Zhou et al. revealed that insulin and IGF-1 increased the proliferation of ISC obtained from obese individuals through the PI3K/Akt pathway. It showed that the canonical ERK pathway was not involved in the increased proliferation. The authors suggested that the abnormal renewal of intestinal epithelial may be associated with inconsistency in signaling between PI3K/Akt and ERK pathways which caused insulin/IGF-1 resistance [104]. A role for PI3K pathway is suggested in regulation of cell cycle and apoptosis through inhibition of cell cycle inhibitors (e.g., phosphorylation of p21Cip1, and p27^kip1^) and inactivation of apoptotic substrates (e.g., Bcl-2-associated death promoter (BAD), procaspase-9) [105], as well as modulating the activity of several transcription factors such as Forkhead family of transcription factors (FoxO), and mTOR [106, 107]. Ostermann er al. showed that HFD-induced obesity increased insulin resistance in intestinal epithelial cells. The impairment in AKT signaling by high-fat diet-induced obesity resulted in FOXO1 transcription factor trap in nucleus, which caused desmosomal cadherin Dsc3 deregulation, c–Myc over-expression, as well as an increase in mitogen activation and tight junction cleavage in colon cells, consequently giving rise to colon tumorigenesis [108]. However, one study demonstrated that obesity/high-fat diet-induced obesity did not affect the Lgr5 + ISCs proliferation and fails to stimulate Akt pathway in ISCs (LRG1, Bmi1 + , Lrig1 +) or progenitor cells. In addition, other proliferation-related signaling, including MAPK and Wnt were not altered. The authors demonstrated that phosphatase and tensin homolog (Pten), a natural inhibitor of Akt pathway, loss is unnecessary for tumor formation in Lgr5 + ISCs. However, Pten deficiency accompanied by Apc loss in Lgr5 + ISCs, synergistically raised proliferation, tumorigenesis, and death rate. Therefore, obesity-induced tumorigenesis in ISC seems to be more dependent on aberrant Wnt/β-catenin than the PI3K-Akt pathway [91]. These inconsistent findings might be the result of variations in duration and composition of the high-fat diets that used.

#### Adiponectin

It is well known that adiponectin decreases in obese people. Dissimilar to the leptin receptor, both adiponectin receptors (AdipoR1 and AdipoR2) and their downstream pathway targets (Appl1, AMPK, and LKB1) were found in ISCs [80]. The literature proposes that adiponectin can stimulate a plethora of downstream signaling like MAPK, PI3K/Akt, AMPK, STAT3, and NF-kB. AMPK stimulates cell proliferation and development through PI3K/AKT/mTOR, thereby inhibiting tumorigenesis [52, 109, 110]. On the contrary, adiponectin administration decreased the number of polyps and tumor mass and increased apoptosis [111–113].

DeClercq et al. demonstrated that in high-fat diet treated mice higher quantity of GFP + stem cells, an increase in Lgr5-GFP + cell division, and a decline apoptosis was observed. Using the colonic organoid culture, the researchers found that adiponectin receptor agonist diminished GFP + stem cell numbers and increased apoptosis in lean mice, but obesity alleviates this effect. In addition, it was shown that obesity may increase stem cell numbers in the intestine and increase tumor formation following decrease of adiponectin signaling [80]. Also, Paneth cells can secret adiponectin, FABP4, and adipsin, which can modulate numerous physiological functions such as metabolic syndrome in autocrine, paracrine, and endocrine manners. The release of adiponectin in Paneth cells may regulated via intestinal tract bacteria *Lactobacillus* through NFkB activation [114]. As was noted, obesity/high-fat diet-induced obesity decreased Paneth cells. Therefore, it is speculated that high-fat diet-induced obesity and obesity-induced bacterial dysbiosis may alter ISC function and numbers through adiponectin drop following Paneth cell reduction.

Altogether, insulin/IGF1 and adiponectin may play a role in predisposing ISCs to tumorigenesis. However, further researches are mandatory to convincingly disclose the effects of the insulin/IGF-1 or adiponectin signaling pathways on ISCs response in obesity regarding tumor initiation.

### Low grade inflammation associated with obesity

Inflammation has been demonstrated to induce the proliferation of intestinal progenitors and stem cells via local inflammation caused by myofibroblasts and immune cells such as dendritic cells, and macrophages [115]. This local cell accumulation causes the release of cytokines, including IL-22, IL-6, IL-17, and TNF-alpha [115, 116], thereby accelerates the growth and proliferation of intestinal progenitors and stem cells. In contrast to the direct effect on ISCs/progenitors, immune cells may also indirectly affect ISCs by regulating other cells within the stem cell niche. For instance, Regulatory T cells play important roles in maintain the homeostasis of lamina propria, a contributor of the IESC niche [117]. Immune cell-derived cytokines, IL1A and IL1B, drive changes in inflammatory responses and extracellular matrix metabolism in myofibroblasts of lamina propria [118].

Latest researches have acknowledged a panel of immune changes associated with an adaptive immune response that are impacted by obesity, such as the elevated CD8 cytotoxic T cell reaction, shifts to Th1/Th17 T cell populations, the reduced number of regulatory T cells, which plays a significant role in additional recruiting M1 macrophages and following inflammation [119, 120].Some studies demonstrated that high-fat diet-induced obesity increases the inflammatory mediators in the intestinal mucosa. For instance, in a model of Genetic TNF-α−/− mice high-fat diet-induced obesity enhanced phosphorylation of GSK3β and entered β-catenin to the nucleus, thereby, increased the levels of Wnt signaling target genes (C-myc, Cyclin D1, and Axin 2). Nevertheless, the deletion of TNF-α diminished these effects—indicating the role of TNF-α induced inflammation on colon carcinogenesis associated with high-fat diet-induced obesity [83]. Similar results were indicated in other studies [121–123]. It should be noted that, the increased level of inflammatory cytokines were different in high-fat diet-induced obesity and genetic-induced obesity as IFNγ and TNF-α elevated in genetically-induced obesity, while IL-6 elevated in diet-induced obesity [124].

It is speculated that inflammation expands colon cell progenitors or stem cells. In this regard, Schwitalla et al. showed that NF-kB activation could stimulate the Wnt pathway and cause reprogramming of Lgr5- enterocytes to acquire stemness features, thereby transforming them to tumor-initiating cells. In fact, NF-kB activation results in binding of RelA/p65 to β-catenin dependent on TNFα and oncogenic K-ras. Given that, oncogenic K-ras can lead to dedifferentiation of Apc-deficient enterocyte through NF-kB activation, in vitro [56]. High-fat diet-induced obesity expanded colon stem cell zone and proliferative zone prior to obesity onset and insulin resistance in a pig model. This increase in the proliferative cells may be owing to the inflammation induced by intestinal bacterial flora dysbiosis. Innate inflammatory markers TLR- 4, NF-kB, LCN-2, and IL6 were highly correlated with proliferative zone, but not the stem cell zone; thereby, affecting the proliferative feature of colonic progenitor cells [125].

Besides, one study indicated that a high-fat diet depleted intestinal eosinophil in mice, causing a defect in gut barrier integrity. However, a high-fat diet did not increase inflammation of the gut. The gene expression of CD11b, CD11c or IL-1β genes were not affected; however, TNF-α and MCP-1 gene expression decreased [126]. Other studies found no connection between the high-fat diet-induced obesity and its predominant fatty acid components with activation of inflammatory signaling (e.g., TNF-α, Infg, the NFκB or the STAT-3) in the APC l/l model mice epithelium or organoids, inflammatory cytokine (IL1, IL6, IL17) receptors in colonic Lgr5 + stem cells [77], and inflammatory cytokine (IL1, IL6, IL17) receptors in colonic Lgr5 + stem cells [80]. HFD has also caused elevated circulating and adipose tissue levels of the prostaglandin E2 (PGE2), a prostaglandin that has increased Lgr5-GFP + cell number and stimulates cell division in Lgr5 organoid cultures [80].

Altogether, the current data indicate that inflammation might have a role in reprogramming of progenitors (or differentiated) cells in the Genetic TNF-α−/− /APC−/− mice models. However, the data that supports the effect of high-fat diet-induced inflammation on ISCs leading to tumorigenesis is contradictory. This inconsistency may be associated with the fact that chronic inflammation is not usually reproducible in mice. It may as well relate to the differences in mice feed between studies [88]. More studies with well-matched diets and a more reliable genetic model are needed to elucidate the effects of obesity-induced inflammation on ISCs for tumor formation.

## Interaction between fats, carbohydrates and gut microbiota

As mentioned above, a high-fat diet can affect the intestinal stem cells or progenitor cells to induce proliferation or reprogram these cells, thereby make them susceptible to tumorigenesis. However, studies have examined the effects of a low-carbohydrate ketogenic diet on ISCs to uncover if these effects are related to dietary fat, or other components such as carbohydrates and proteins. A new study demonstrated that fat and sugar play different roles in intestinal homeostasis. In fact, a high-fat-diet-induced ketogenesis enhances ISCs function, self-renewal, and epithelial regeneration via beta-hydroxybutyrate (βOHB), which enhance Notch pathway in ISCs. Nonetheless, a high sugar supplement diminished these effects by decreasing βOHB production [127]. The presence of glucose may also partially explain the contrary results of treatment with βOHB in cancer cells [128, 129]. Therefore, the presence of carbohydrates into a high-fat diet and the effect of interaction between them on ISC function should always be considered [127].

A recent study found that constitutive activation of a glycolytic and non-oxidative processes of carbohydrate metabolism might make ISCs susceptible to tumorigenesis. The Authors indicated that, in various mouse intestinal cancer models, glycolysis and related pathways could promote tumor formation. It was proposed that the decline in pyruvate oxidation in mitochondria might be necessary for tumor formation, and constitutive enforcement of high uptake of pyruvate by mitochondria was enough to inhibit tumorigenesis entirely. Mitochondrial pyruvate carrier1 (*MPC1*) is a gene involved in pyruvate transport, required for oxidation metabolism, and is negatively associated with the activity of the Wnt/b-catenin signaling in human normal and adenomatous colon. The deficiency of Apc (which along with further Wnt/b-catenin pathway activity) enforces the decline of the *MPC1* gene expression and establishes a glycolytic metabolic phenotype. These results indicate the decisive role of pyruvate in regulation of ISC proliferation [130].

In fact, absorptive enterocytes and secretory enteroendocrine can sense the nutrients and capability of others cell to absorb nutrients is still unidentified. Researches indicate that enterocytes, Paneth, and goblet cells can absorb the fructose [131], however, Lgr5_-ISC cells cannot sense fructose perhaps because of low GLUT5 and ketohexokinase (KHK) levels [131]. On the other hand, Paneth cells metabolize glucose by glycolysis and secrete the produced lactate into their surrounding and enter the adjacent stem cells. Within the ISC, lactate is then converted to pyruvate by lactate dehydrogenase which consequently enters TCA as Acetyl CoA [132]. As previously discussed, high-fat diet-induced obesity is associated with a decrease in Paneth cell numbers in mouse intestine as well as activation of Wnt/b-catenin. Therefore, it might cause a decline in pyruvate uptake by the ISCs and might activate the Wnt/b-catenin, thereby establishing a glycolytic metabolic state and initiate tumor formations. It was also demonstrated that the lactate produced by Lactic acid producing bacteria (LAB) was dependent on a lactate-specific G‐protein coupled receptor 81 (GPR81) on membrane cells, and induced the Wnt/b-catenin pathway in Paneth cells and intestinal stromal cells thereby expanding proliferation of ISCs, goblet cells, and Paneth cells [133]. In addition, lactate stimulated porcupine (PORCN) signaling in the Paneth cells and intestinal stromal cells resulting in Wnt3 expression to maintain ISCs [133]. Besides, high-fat diet reduces the number of lactic acid bacteria in the gut tract [134]. Therefore, it is speculated that in the colon, where Paneth cells are absent, the high-fat diet decreased pyruvate uptake of colonic ISCs via a decrease in lactic acid bacteria, thus increasing tumor formation. Together, the interaction between high fat diet with carbohydrates and microbiota plays an important role in distortion of carbohydrate metabolism toward a glycolytic phenotype and elevated FA uptake into ISCs, leading to increased susceptibility of stem cells to tumor formation.

## Complexities and limitations

As could be noted, the findings are controversial. There are several differences between the various studies that could explain the inconsistencies observed. For example, the type of diet and obese genetic model used in studies are not consistent. Comparing the type of high-fat diet (HFD), the difference in the amount of fat in the diet or the time and duration of treatment differs; [60% HFD ([77], 80, 79, 135, 75) or 45% HFD; [22], 91], [low-fat ([80], 135, 22) or chow ([77], 79, 75], as well as [2–4 months; [80], 79; 135, 75), 5–6 months [22] or 7–14 month [77]]. Nutrient components of feed also may vary in different studies. Many studies used a regular rodent chow-based feed as the control mice group. Although mice that consume this standard diet do remain leaner than their HFD counterparts, it harbors a poor concentration of special micronutrients and macronutrients as well as higher vitamin D and fiber and the content of these parts may also differ significantly among feeds [91]. Many of these components play an important role in cell biological activity, particularly in the intestinal hemostasis, and may markedly affect the experiment [91]. For instance, a high-fat diet drives LGR5 expression, ISC transformation, and tumorigenesis in mice colon cancer via an increase in vitamin A-bound serum retinol-binding protein 4-stimulated by retinoic acid 6 (RBP4-STRA6) signaling pathway [136]. Dietary cholesterol can induce tumorigenesis mediated by phospholipid remodeling [82]. Dietary methionine-derived SAM in Drosophila intestine modulates tumorigenesis through regulation of the cytokine upd3 expression (137). Fiber can inhibit ISC division through production of butyrate and microbiota profile alteration [138]. Dietary glutamate increases ISC proliferation through modulating calcium signaling in Drosophila [83]. It is also indicated that in mice treated with western like diet (WD), characterized by low vitamin D3 and calcium crypt cell differentiation and Wnt pathway was disturbed in colonic epithelium and Lgr5 + ISC was reduced. In addition, a lower vitamin D3 or calcium level in WD raised the DNA mismatch repair pathway in Lgr5 + cells. WD also induced Bmi1 + cells to mimic stem cells function in intestine homeostasis and tumorigenesis [139]. Therefore, the specific contribution of HFD/obesity in studies with standard diet as a control, especially in the intestine, should be interpreted with prudence due to the obvious variation in nutritional values among the feeds [91].

Another issue that should be addressed is the use of genetic mouse models used in these studies as the model develops adenoma in the small intestine and often do not progress to invasive carcinoma. In humans, small bowel cancers are rare and most of the human intestinal cancer occurs in the colon and rectum [140, 141]. Moreover, many conditions that accelerate colorectal formation in humans, such as chronic inflammation, do not generally occur in mouse models [88, 142].

## Conclusion

The obesity or high-fat diet-induced obesity increases the ISC pool and induces biological modulation in these cells. It is assumed that this abnormality may play an important role in the pathogenesis of intestinal cancer. However, the exact effect of obesity/ high-fat diet-induced obesity on ISCs that link obesity to cancer is not well elucidated because of the limited mice model of cancer and the difference in mice feed that was used in the previous researches. Besides, the presence of both carbohydrates and fats should be considered for observed effect on ISCs. In fact, the interaction between fat and carbohydrate on gut microbiota and ISCs niche appears to be involved in the pathogenesis of intestinal cancer. Altogether, it is expected that forthcoming investigations on how obesity/high-fat-diet-induced obesity orchestrates gut homeostasis and cell metabolism provide new documents for the prevention of CRC initiation and development. On the other hand, it is necessary to more clearly elucidate the role of different high-fat diets (including high fat and carbohydrates, and ketogenic diet) on ISC behavior, especially the metabolic changes in obesity/high-fat diet-induced obesity state, and their susceptibility to tumorigenesis.

## Data Availability

Not applicable.

## Abbreviations

βOHB: Beta-hydroxyl butyrate
WSD: Western diet
TCA: Tricarboxylic acid cycle
mTOR: Mammalian target of rapamycin
ISC: Intestinal stem cell
EEC: Enteroendocrine cells
HFD: High fat diet
LFD: Low fat diet
FXR: Farnesoid X receptor
PPAR: Peroxisome proliferator-activated receptors
